# Functional deletion of neuropeptide Y receptors type 2 in local synaptic networks of anteroventral BNST facilitates recall and increases return of fear

**DOI:** 10.1038/s41380-020-0846-x

**Published:** 2020-07-24

**Authors:** Julia Constance Bartsch, Sara Jamil, Jasmin Remmes, Dilip Verma, Hans-Christian Pape

**Affiliations:** Institute of Physiology I, Westfälische Wilhelms-University, D-48149 Münster, Germany

**Keywords:** Neuroscience, Physiology, Psychiatric disorders

## Abstract

Return of previously extinguished fear memories presents a major hurdle in treatment of fear-related disorders. Neuropeptide Y receptors type 2 (Y2R) in the bed nucleus of stria terminalis (BNST) seem to play a crucial role in modulation of remote fear memories. Here, we targeted Cre-channelrhodopsin-2 to defined subregions of BNST or central amygdala (CeA) in floxed Y2R mice (Y2^lox/lox^) for functional deletion of Y2R. We combined fear training and behavioral studies in vivo with optogenetic-electrophysiological analysis of BNST synaptic network activity ex vivo, in order to identify regional and cellular specificities of Y2R influence. Deletion of Y2R in the ventral section of anterior BNST (BNSTav) did not affect fear acquisition, but increased conditioned fear during recall and extinction learning, and aggravated remote fear return. By contrast, deletion of Y2R in the dorsal section of anterior BNST (BNSTad) or CeA did not influence acquisition, extinction or return of fear memories. Ex vivo optogenetic-electrophysiological analysis revealed Y2R-expressing local GABAergic inhibitory networks in BNST, both within (intraregional) and in-between (inter-regional) BNST subregions. Stimulation of Y2R resulted in a presynaptically mediated reduction of GABAergic responses, which did not differ between intraregional but predominantly affected inter-regional connections from BNSTav to BNSTad. Moreover, deletion of Y2R decreased the excitation/inhibition balance in BNSTav neurons, suggesting a regulatory influence of endogenous NPY via intraregional GABAergic microcircuits. This study reveals Y2R within local GABAergic networks in BNST as key elements in facilitating extinction and reducing return of remote fear memories, suggesting a potential avenue for translational purposes.

## Introduction

Fear memory traces are formed instantaneously and remain robust throughout an organism’s life span [1, 2], supporting behavioral adaptation under threat. However, fear memories which fail to update their predictive values are no longer efficient and underlie many of the symptomatologies associated with a dysregulated fear response system, such as posttraumatic stress disorder (PTSD). The bed nucleus of stria terminalis (BNST), part of the extended amygdala, has recently gained interest as a site for integration of signals relating to states of anxiety and stress-mediated disorders [3–5]. Preclinical models identify synaptic circuits within BNST to process ambiguous threat signals and responses to cue reminders of sustained threat [6, 7], implicating an involvement in long-term fear regulation [8]. Of particular interest is a recent study highlighting neuropeptide Y (NPY), a 36-amino-acid peptide known for its anxiolytic properties [9], as potential mediator of long-term fear regulation within anteroventral BNST (BNSTav) [10]. Pharmacological stimulation of NPY receptor type 2 (Y2R) resulted in a suppression of fear return, whereas blockade of Y2R promoted return of fear at remote stages after extinction training. The presynaptic nature of Y2R [9, 11], the reported influence of NPY on fear regulation in central amygdala (CeA) projecting to anterior BNST [12], and the heterogeneous organization of BNST and neuropeptides present within its nuclei [8, 13], all precluded conclusions regarding the underlying circuits and mechanisms.

Therefore, in the present study, we employed Cre-mediated functional deletion of Y2R in defined subregions of BNST or CeA of Y2^lox/lox^ mice, and combined fear training and behavioral studies in vivo with optogenetic-electrophysiological analysis of BNST synaptic network activity ex vivo, in order to identify regional and cellular underpinnings of Y2R-mediated behavioral effects.

## Materials and methods

For detailed description of methods see Supplementary Material and Methods.

### Animals

All experiments were carried out in accordance with the European Committees Council Directive (2010/63/EU of the European Parliament) and were approved by the local authority (LANUV). Y2^lox/lox^ mice [14] were used for all experiments. Animals were randomly allocated to the different experimental groups. After surgical procedure, mice were single-housed, maintained on ad libitum diet in a 12 h light–dark cycle.

### Stereotaxic surgery and viral infusion

Adult male mice (8–10 weeks) were anesthetized with pentobarbital (50 mg/kg i.p; Narkoren, Pharmacy Universitätsklinikum Münster, Germany), and injected with carprofen (5 mg/kg s.c.; Rimadyl, Pfizer, Berlin, Germany) before stereotaxic surgery (Model 902, David Kopf Instruments, California, USA) [15]. For BNSTav, guide cannulas (26GA, 4 mm, PlasticsOne, Virginia, USA) were implanted bilaterally (AP, +0.4 mm from Bregma; ML, ±2.0 mm from the midline; DV, 4.4 mm at an angle of 14°), and viral injections were performed after 7–10 days of recovery. For BNSTad (AP, +0.4 mm; ML, ±1.8 mm; DV, 3.8 mm) and CeA (AP, −1.6 mm; ML, ±3.1 mm; DV, 3.8 mm), viral injections were performed during the surgical procedure itself. AAV-Syn-ChR2-eYFP (5.6 × 10^12^ copies/ml) or AAV-Syn-Cre-ChR2-eYFP (1.56 × 10^13^ copies/ml) were infused bilaterally at a rate of 50 nl/min in a total volume of 150 nl using MicroSyringe pump (UMP3) and controller (Micro4; World Precision Instruments, Sarasota, Florida, USA) connected via 5 µl Hamilton syringe to stainless steel cannulas (33GA, 5 mm). Between 3 and 4 weeks were allowed before commencement of all experiments for adequate transfection of the viral vectors after injections.

### Pharmacological procedures

In a subset of animals with BNSTav cannula implantation, the Y2R agonist NPY_3–36_ (PolyPeptide Laboratories, Strasbourg, France), the Y2R antagonist JNJ-5207787 (Tocris Bioscience, Wiesbaden, Germany), or saline (0.9% NaCl; pH 7.4) were infused bilaterally into BNSTav at a rate of 100 nl/min in a total volume of 300 nl, 20 min prior to extinction training [10, 16]. NPY_3–36_ and JNJ-5207787 were prepared at the day of the experiment from stock solutions at 200 nM.

While NPY_3–36_ has a potent affinity for Y2R, it also does have an affinity for Y5R [17]. However, within the BNSTav there is little evidence of functional Y5Rs. This makes NPY_3–36_ a good agonist for the present study. JNJ-5207787 is a potent and selective Y2R antagonist devoid of affinity for Y1, Y4, and Y5 receptors [18]. Both Y2R ligands have previously been used for in vivo pharmacological experiments in anterior BNST of fear-conditioned mice [10, 16]. Therefore, we chose these ligands to maintain between-study consistency and to replicate the in vivo findings from nongenetically modified mice to the genetically modified Y2R^lox/lox^ mice.

### Verification of injection site and deletion of Y2R

Viral transfection sites were verified by examining the expression of eYFP in 250 μm brain slices comprising the entire BNST. Only animals which showed regional-specific bilateral transfections within BNSTav, BNSTad, or CeA were included for analysis. In pharmacological experiments, position of cannula tips was verified in histological sections. Tissue was prepared as described in [10]. Only animals in which tissue injury was located at or just above BNSTav, were included in analysis. In a subset of animals, BNSTav and BNSTad were laser-dissected (16 μm slices), and specificity of Y2R deletion was verified through PCR assessment of Cre-mediated recombination of Y2R (Supplementary Fig. 1). The expression of Y2R mRNA (16 μm slices) in BNSTav was further quantified using RNAscope (ACDbio, CA, USA; Supplementary Figs. 2 and 3).

### Behavioral paradigm

Mice were trained in a differential fear conditioning paradigm as previously described [10] with fear conditioning on day 1 and fear extinction and remote fear recall conducted in a separate context on day 2 and day 16, respectively. Freezing to the CS+ presentations was averaged across three consecutive CS+ presentations (3CS+ block) for extinction training and remote fear recall tests. Freezing scores were analyzed independently by three experienced experimenters.

### Ex vivo electrophysiology

On day 17, 24 h after remote fear memory retrieval, mice were decapitated under deep isoflurane anesthesia (2.5% in O_2_, Abbott, Wiesbaden, Germany) and coronal slices (250 μm) containing the anterior BNST were prepared as previously described [19]. To study the intrinsic connectivity of BNST neurons, channelrhodopsin-2 (ChR2)-assisted “paired” patch-clamp recordings were performed. A ChR2-eYFP-expressing BNST neuron was activated by scanning a somatic region (~3 µm diameter) with a 473 nm laser (~30 ms scan time). Optogenetically activated inhibitory postsynaptic currents (oIPSCs) in neighboring BNST neurons were recorded in whole-cell voltage-clamp mode at a holding potential of 0 mV using a Cs-based internal solution [20]. oIPSC amplitudes were analyzed during baseline, in the presence of the Y2R agonist Peptide YY 3–36 (PYY_3–36_, Genscript, Piscataway, NJ, USA; bath-applied at 200 nM for 10 min), and after washout. In some experiments, postsynaptic inhibitory currents (eIPSCs) were evoked by a bipolar tungsten stimulation electrode placed dorsally to the recording electrode in the surrounding neuropil. To examine excitation/inhibition (E/I) ratios, individual BNST neurons were first voltage-clamped at −65 mV, and then at 0 mV to record spontaneous excitatory and inhibitory synaptic currents (sEPSCs and sIPSCs), respectively. Electrophysiological data were analyzed offline with Clampfit (Molecular Devices Corporation, Sunnyvale, CA, USA) or Mini Analysis (Synaptosoft Inc., Fort Lee, NJ, USA).

### Statistical analysis

Sample size was based on prior studies [7, 10]. All datasets were assessed for outliers using the Grubbs’ test (significance level *p* < 0.05) or studentized residuals scores (*z* = ±3). Assumptions for analysis of variance (ANOVA) were tested. In case of sphericity violations, Greenhouse–Geisser corrections were used. Data are presented as mean, mean difference ± standard error of the mean or box and whisker plots (box: 25th to 75th percentiles, whiskers 5th and 95th percentiles), and considered significant at *p* < 0.05. Statistics were conducted using SPSS (IBM SPSS Statistics, version 24) or GraphPad Prism (GraphPad Software, San Diego, CA). Experiments were performed in a randomized fashion.

#### In vivo

In statistical analysis of extinction and remote recall, each data point, except the pre-CS, represents a mean of freezing percentages for three consecutive CS+ presentations (3CS+ block). Mixed model two-way ANOVA with freezing percentages as dependent variable and viral injection groups and time as between-subject and within-subject factors, respectively, was used to analyze freezing behavior between groups followed by post hoc Bonferroni for statistically significant interactions. Wilcoxon*–*Mann*–*Whitney test was used for statistical analysis of Y2R expression in DAPI-positive cells.

#### Ex vivo

Statistical significance was tested using either mixed model two-way ANOVA followed by post hoc Sidak’s multiple comparison test, one-way repeated measures ANOVA followed by post hoc Dunnett’s multiple comparison test, or Kruskal–Wallis test followed by post hoc Dunn’s multiple comparison test. Numbers given in text (x/y) refer to numbers of neurons (x) recorded in different animals (y).

## Results

### Deletion of Y2R in BNSTav increases fear during extinction learning and promotes recall of conditioned and remote fear

**Fig. 1:**
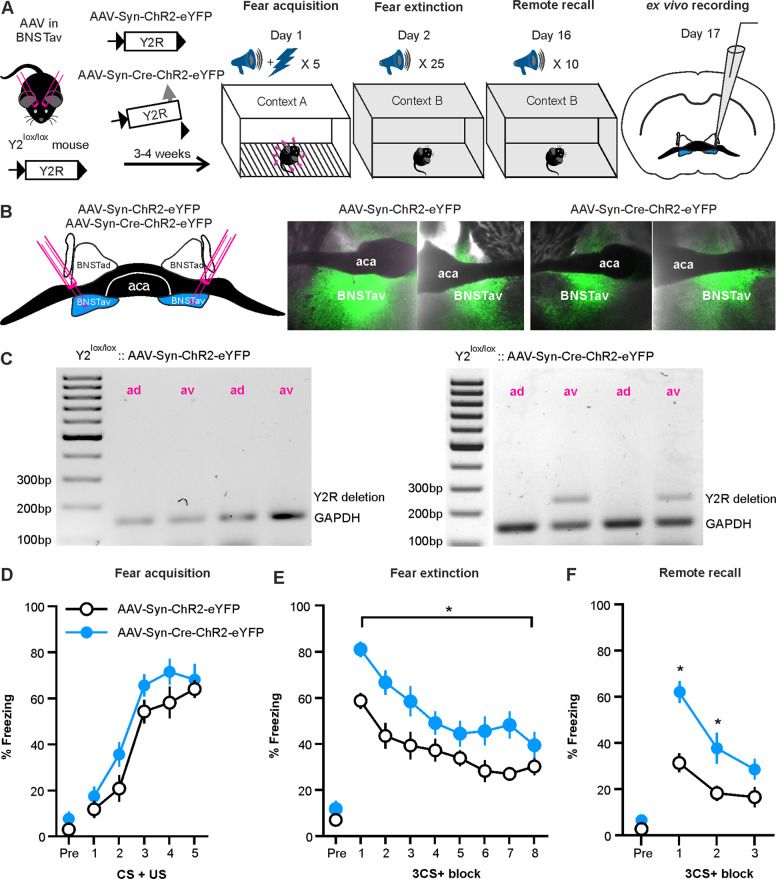
Cre-mediated deletion of Y2R within BNSTav and the effects on acquisition, extinction, and remote recall of fear. **a** Schematic representation of the experimental paradigm depicting the genetic model employed for Cre-mediated deletion of Y2R in BNSTav and the behavioral paradigm with timeline. **b** A representative illustration of the local transfection site within the BNSTav and ex vivo brain sections expressing the eYFP fluorescence localized within the transfected BNSTav region after injection of AAV-syn-ChR2-eYFP and AAV-Syn-Cre-ChR2-eYFP. **c** Representative agarose gel electrophoresis photograph showing PCR results for samples from both the AAV-Syn-ChR2-eYFP-injected (left) and AAV-Syn-Cre-ChR2-eYFP-injected mice (right). Cre-mediated recombination results in a 250 bp PCR product. Note that the recombination-specific product is present only in BNSTav samples from Cre-injected mice and absent in BNSTad samples used as an internal control. **d** Fear acquisition across time. Each data point represents freezing percentages for a single CS + US presentation (group: *F*_(1,24)_ = 3.92, *p* = 0.059 partial η^2^ = 0.140; time: *F*_(5,120)_ = 82.52, *p* < 0.001, partial η^2^ = 0.775; interaction: *F*_(5,120)_ = 0.66, *p* = 0.673, partial η^2^ = 0.026). **e** Extinction learning across time. Each data point represents freezing percentages averaged across three consecutive CS+ blocks. Statistically significant group differences exist across time, with Cre-injected mice showing a higher freezing level (group: *F*_(1,24)_ = 16.92, *p* < 0.001, partial η^2^ = 0.414; time: *F*_(8,192)_ = 38.42, *p* < 0.001, partial η^2^ = 0.616; interaction: *F*_(8,192)_ = 1.06, *p* = 0.395, partial η^2^ = 0.042) and a delayed learning of extinction compared to control mice (3rd 3CS+ block compared to 4th 3CS+ block: 21.56 ± 4.74%, *p* = 0.016; 29.26 ± 2.45%, *p* < 0.001. Post hoc analysis of the 8th CS+ block reveals no differences between groups *p* > 0.05. **f** Fear recall at remote time. Each data point represents freezing percentages averaged across three consecutive CS+ blocks. A significant difference between freezing levels exist within the groups at different time points (interaction: *F*_(3,72)_ = 5.12, *p* < 0.005, partial η^2^ = 0.176) where Cre-injected mice show a much higher level of freezing at the 1st and the 2nd 3CS+ block (*F*_(1,24)_ = 24.21, *p* = 0.001, partial η^2^ = 0.502; *F*_(1,24)_ = 8.01, *p* = 0.009, partial η^2^ = 0.25; 30.51 ± 6.19%, *p* < 0.001; 19.51 ± 6.89%, *p* < 0.005). Post hoc test of baseline freezing percentages prior to the onset of CS + US in fear acquisition, or CS+ in either fear extinction or remote recall tests do not reveal differences between groups (all *p* > 0.05).

In order to investigate the role of local Y2R within the BNSTav, Y2^lox/lox^ mice were bilaterally injected with either AAV-Syn-Cre-ChR2-eYFP (*n* = 11) for Cre-mediated deletion of Y2R, or with AAV-Syn-ChR2-eYFP (*n* = 15) as control. Mice were exposed to a differential fear conditioning paradigm [10], and expression of fear was assessed during acquisition (day 1), extinction (day 2) and remote recall of fear (day 16). On the following day, BNST slices were prepared and used for subsequent ex vivo electrophysiological experiments (Fig. 1a). Specificity of transfection sites was histologically verified through eYFP expression in ex vivo slices (Fig. 1b) and through detection of Cre-mediated Y2R recombination via PCR in a subset of animals (*n* = 6/group; Fig. 1c). Furthermore, expression of Y2R mRNA was detected using RNAScope (Supplementary Fig. 2) and quantified using ImageJ, in control and Cre-injected mice. The percentage of DAPI-positive nuclei coexpressing Y2R mRNA was significantly reduced in Cre-injected mice (*p* < 0.01) (Supplementary Fig. 3).

To assess for behavioral correlates of Y2R deletion, CS+-evoked freezing levels were statistically compared between the two groups of animals using mixed model ANOVA for repeated measures. All mice acquired fear, and no group differences were found between Cre-injected mice and controls (Fig. 1d). In extinction learning, Cre-injected animals showed a higher level of freezing across time (Fig. 1e). In order to analyze the rate of extinction learning, freezing percentages at each subsequent 3CS+ blocks were statistically compared to the freezing percentage of the first 3CS+ block within each injection group. Significant reduction in freezing occurred by the 3rd 3CS+ block in control and the 4th 3CS+ block in Cre-injected mice, and no differences existed between groups at the last 3CS+ block of extinction training, suggesting that all groups had acquired extinction by the last 3CS+ block (Fig. 1e). Fear recall at remote time point was significantly different between groups, such that the Cre-injected mice displayed higher freezing in response to both the first and the second 3CS+ block, indicating a higher level of remote fear recall as compared to controls (Fig. 1f).

In order to validate the Y2^lox/lox^ mouse model, with respect to pharmacological results obtained with Y2R manipulation in BNST of C57BL/6N mice [10], we injected Y2R agonist (NPY_3–36_; *n* = 11), Y2R antagonist (JNJ-5207787; *n* = 9), and saline (0.9% NaCl; *n* = 10) bilaterally into BNSTav in a separate group of Y2^lox/lox^ mice (Supplementary Fig. 4). All mice acquired fear and were subsequently divided into three equally performing groups according to freezing levels (Supplementary Fig. 4B). During fear extinction, JNJ-5207787-infused Y2^lox/lox^ mice showed a significantly higher freezing level across time compared to saline-injected animals, whereas NPY_3–36_-infused animals were not different from controls (Supplementary Fig. 4C). During recall of remote fear, a significant group difference was found depending on time. In JNJ-5207787-infused animals, freezing was higher compared to controls during the first 3CS+ block and remained high during the last 3CS+ block, whereas NPY_3–36_-infused animals displayed lower freezing compared to controls during the first block of CS+ presentation (Supplementary Fig. 4D).

### Deletion of Y2R in BNSTad does not affect acquisition, extinction, or recall of fear

**Fig. 2:**
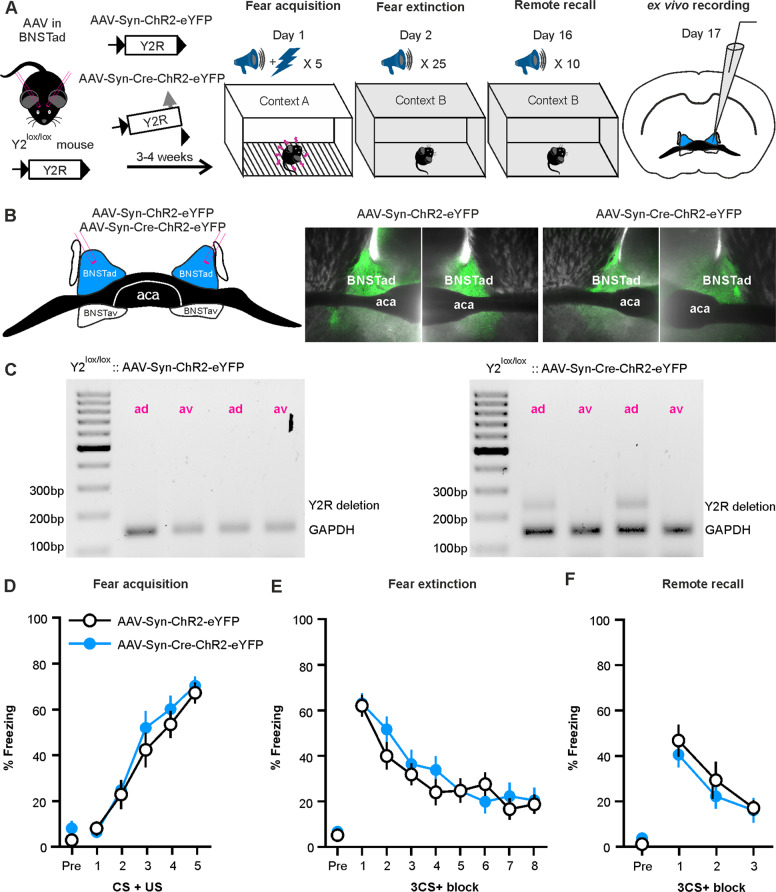
Cre-mediated deletion of Y2R within BNSTad and the effects on acquisition, extinction, and remote recall of fear. **a** Schematic representation of the experimental paradigm depicting the genetic model employed for Cre-mediated deletion of Y2R in BNSTad and the behavioral paradigm with timeline. **b** A representative illustration of the local transfection site within the BNSTad and ex vivo brain sections expressing the eYFP fluorescence localized within the transfected BNSTad region after injection of AAV-Syn-ChR2-eYFP and AAV-Syn-Cre-ChR2-eYFP. **c** Representative agarose gel electrophoresis photograph showing PCR products from both the AAV-Syn-ChR2-eYFP-injected (left) and the AAV-Syn-Cre-ChR2-eYFP-injected mice (right). Cre-mediated recombination results in a 250 bp PCR product. Note that the recombination-specific product is present only in BNSTad samples from Cre-injected mice and absent in BNSTav samples used as an internal control. **d** Fear acquisition across time. Each data point represents freezing percentages for a single CS + US presentation (group: *F*_(1,27)_ = 0.29, *p* = 0.591, partial η^2^ = 0.011; time: *F*_(5,135)_ = 80.13, *p* < 0.001, partial η^2^ = 0.748; interaction: *F*_(5,135)_ = 0.31, *p* = 0.536, partial η^2^ = 0.030). **e** Extinction learning across time. Each data point represents freezing percentages averaged across three consecutive CS+ blocks (group: *F*_(1,27)_ = 1.19, *p* = 0.586, partial η^2^ = 0.011; time: *F*_(8,216)_ = 38.99, *p* < 0.001, partial η^2^ = 0.591; interaction: *F*_(8,216)_ = 1.19, *p* = 0.320, partial η^2^ = 0.042). No significant differences were found between BNSTad and BNSTav control groups for freezing in fear extinction (group: *F*_(1,26)_ = 1.556, *p* = 0.223; time: *F*_(8,208)_ = 35.719, *p* < 0.001; interaction: *F*_(8,208)_ = 0.970, *p* = 0.461) and remote recall tests (group: *F*_(1,26)_ = 2.194, *p* = 0.151; time: *F*_(2.257,58.678)_ = 35.142, *p* < 0.001; interaction: *F*_(2.257,58.678)_ = 2.542, *p* = 0.082). **f** Fear recall at remote time. Each data point represents freezing percentages averaged across three consecutive CS+ blocks (group: *F*_(1,27)_ = 0.27, *p* = 0.605, partial η^2^ = 0.010; time: *F*_(3,81)_ = 38.38, *p* < 0.001, partial η^2^ = 0.587; interaction: *F*_(3,81)_ = 1.19, *p* = 0.570, partial η^2^ = 0.024). Post hoc test of baseline freezing percentages prior to the onset of CS + US in fear acquisition, or CS+ in either fear extinction or remote recall tests do not reveal differences between groups (all *p* > 0.05).

The role of local Y2R within the BNSTad was assessed next by injecting AAV-Syn-Cre-ChR2-eYFP (*n* = 16) and AAV-Syn-ChR2-eYFP (*n* = 13) in Y2^lox/lox^ mice. Experimental paradigms (Fig. 2a), verification of transfection specificity (Fig. 2b, c), and behavioral assessment of fear were conducted as for BNSTav. Animals of all groups acquired (Fig. 2d), extinguished (Fig. 2e), and recalled remote fear (Fig. 2f), without differences occurring between groups at any experimental time point during CS+-evoked freezing. Notably, two outliers in the control group displayed baseline freezing at *z* = 3.23 and *z* = 3.34 and were included because ANOVA tests with and without the outliers were not statistically different.

No significant differences were found between BNSTad and BNSTav control groups for freezing in fear extinction (compare Figs. 1e and 2e).

### Deletion of Y2R in CeA does not affect acquisition, extinction, or remote recall of fear

Finally, we targeted the CeA in Y2^lox/lox^ mice, through bilateral injection of AAV-Syn-Cre-ChR2-eYFP (*n* = 7) and AAV-Syn-ChR2-eYFP (*n* = 7), using procedures as described above (Supplementary Fig. 5). No statistically significant difference between groups was observed in fear acquisition, extinction learning, or remote fear recall. Regardless of groups, animals showed similar freezing levels across all behavioral tests.

### Y2R activation decreases GABAergic signaling in intraregional BNST microcircuits

**Fig. 3:**
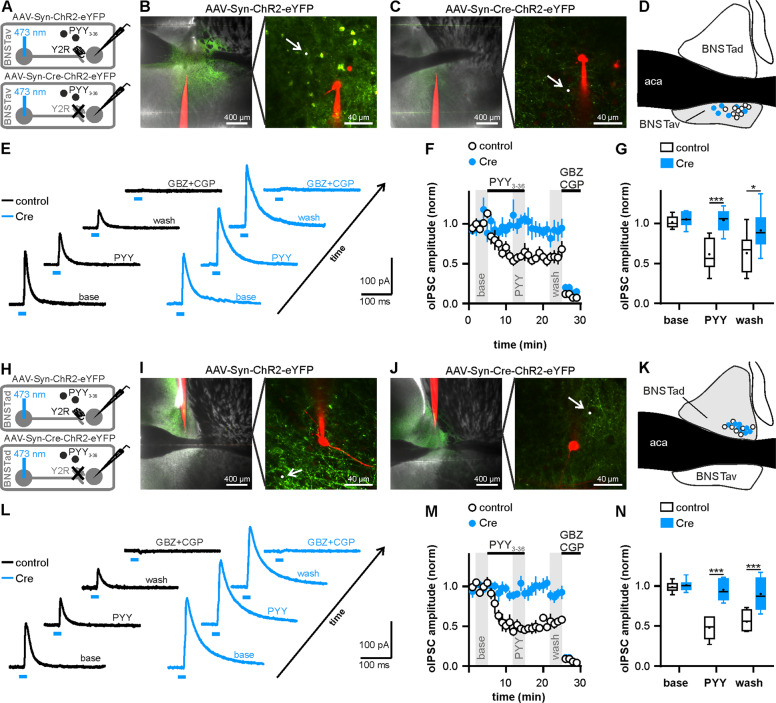
Y2R activation decreases GABAergic signaling in intraregional BNST microcircuits. **a** Scheme of experimental approach to assess potential inhibitory synaptic connections between BNSTav neurons and their modulation by Y2R activation. BNSTav neurons were transduced using either AAV-Syn-ChR2-eYFP or AAV-Syn-Cre-ChR2-eYFP and optogenetic experiments were performed 6 weeks later. **b**, **c** Presentation of a typical recording site in BNSTav and placement of the scanning ROI (white circle) on a ChR2-expressing neighboring neuron of the recorded neuron (red, Alexa594-filled). **d** Localization of recorded neurons outlined in a schematic coronal section of the anterior BNST. White symbols denote cells recorded in slices from Y2^lox/lox^ mice injected with AAV-Syn-ChR2-eYFP (termed “control” in the following) and blue symbols refer to cells recorded in slices from Y2^lox/lox^ mice injected with AAV-Syn-Cre-ChR2-eYFP (termed “Cre” in the following). **e** Representative traces of oIPSCs recorded in BNSTav neurons at a holding potential of 0 mV (averaged from 9 responses during baseline, PYY_3–36_ bath application, washout, and during addition of GABA_A/B_ antagonists). Blue bars indicate the light stimulus. **f** Time course of the mean normalized oIPSCs amplitudes at BNSTav to BNSTav synapses. Notably, Y2R activation reduced oIPSC amplitudes at BNSTav to BNSTav synapses in control mice. Gray boxes indicate time points used for statistical analysis of the light-evoked responses during the different recording conditions (baseline, PYY_3–36_ application, washout; control: *n* = 9/6, Cre: *n* = 6/5). **g** Quantification of the mean normalized oIPSC amplitudes (interaction: *F*_(2,26)_ = 5.08, *p* < 0.05, time: *F*_(2,26)_ = 10.40, *p* < 0.001, group: *F*_(1,13)_ = 13.61, *p* < 0.01, ***p < 0.001, *p < 0.05 by post hoc Sidak). **h** Scheme of experimental approach to assess potential inhibitory synaptic connections between BNSTad neurons and their modulation by Y2R activation. BNSTad neurons were transduced using either AAV-Syn-ChR2-eYFP or AAV-Syn-Cre-ChR2-eYFP and optogenetic experiments were performed 6 weeks later. **i**, **j** Presentation of a typical recording site in BNSTad and placement of the scanning ROI (white circle) on a ChR2-expressing neighboring neuron of the recorded neuron (red, Alexa594-filled). **k** Localization of recorded neurons in BNSTad. **l** Representative traces of oIPSCs recorded in BNSTad neurons at a holding potential of 0 mV during the different recording conditions. Blue bars indicate the light stimulus. **m** Time course of the mean normalized oIPSCs amplitudes at BNSTad to BNSTad synapses (control: *n* = 7/4, Cre: *n* = 8/7). Y2R activation reduced oIPSC amplitudes at BNSTad to BNSTad synapses in control mice. **n** Quantification of the mean normalized oIPSC amplitudes (interaction: *F*_(2,26)_ = 15.02, *p* < 0.001, time: *F*_(2,26)_ = 28.21, *p* < 0.001, group: *F*_(1,13)_ = 41.19, *p* < 0.001, ***p < 0.001 by post hoc Sidak).

In a complementary line of experiments, we investigated the impact of Y2R modulation on BNST microcircuits using ChR2-assisted “paired” patch-clamp recordings in brain slices prepared on the day after remote fear recall. A ChR2-eYFP-expressing BNST neuron was activated by scanning a small circular region of interest (~3 µm diameter) on its soma with a 473 nm laser (~30 ms scan time, Fig. 3a–d, h–k). Focal photoactivation reliably (apparent connectivity ~92%, 59 out of 64 tested pairs) evoked inhibitory postsynaptic currents (oIPSCs) in neighboring neurons recorded at 0 mV in voltage-clamp mode in both BNSTav and BNSTad (Fig. 3e, l). Light-activated postsynaptic currents occurred within laser scan time, were stimulus-locked with little onset latency jitter, and sensitive to GABA receptor antagonists (10 μM Gabazine, 2.5 µM CGP55845; Fig. 3f, m). Bath application of the Y2R agonist PYY_3–36_ (200 nM) for 10 min significantly reduced oIPSC amplitudes in control mice in both BNSTav and BNSTad neurons (Fig. 3e–g, l–n), indicating a Y2R-mediated modulation of inhibitory intraregional BNST microcircuits. This modulation was lost in Cre-injected mice, corroborating the functional deletion of Y2R in intraregional inhibitory BNST microcircuits (Fig. 3e–g, l–n). These results indicate that PYY_3–36_ can decrease GABAergic signaling at intraregional BNST synapses via Y2R activation on local BNST interneurons.

### Predominantly inter-regional inhibitory BNSTav to BNSTad microcircuits are Y2R-modulated

**Fig. 4:**
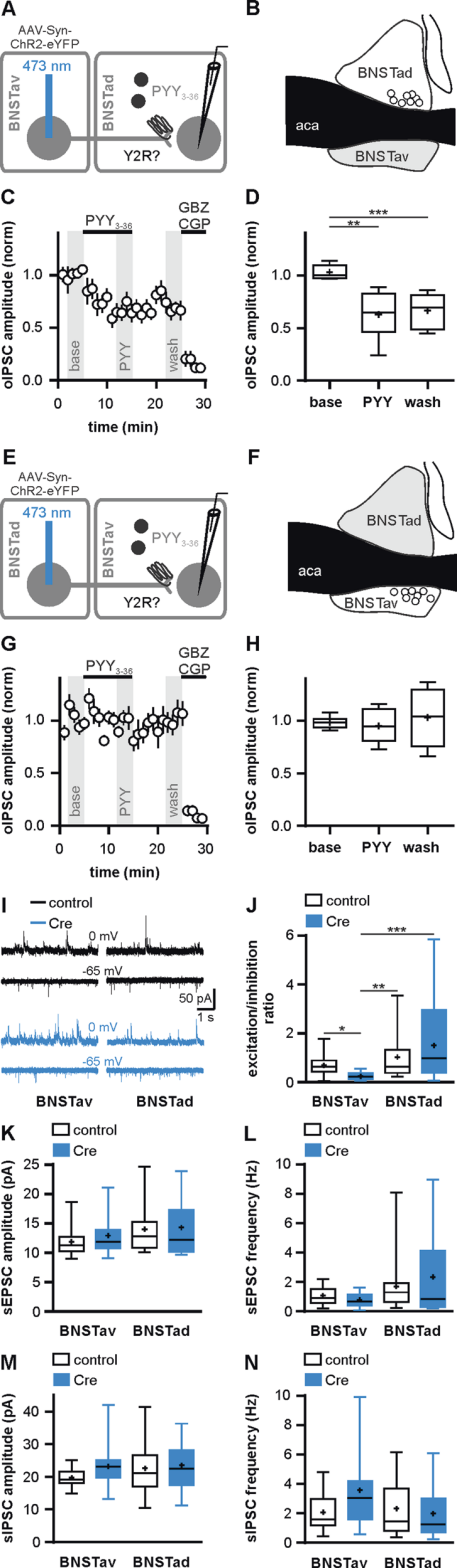
Inter-regional inhibitory BNST microcircuits are differentially regulated by Y2R. **a** Scheme of experimental approach to assess potential inhibitory synaptic connections between BNSTav and BNSTad neurons and their modulation by Y2R activation. BNSTav neurons were transduced using AAV-Syn-ChR2-eYFP and optogenetic experiments were performed 6 weeks later. **b** Localization of recorded neurons in BNSTad. **c** Time course of the mean normalized oIPSCs amplitudes at BNSTav to BNSTad synapses (*n* = 8/5). Y2R activation reduced oIPSC amplitudes at BNSTav to BNSTad synapses in control mice. **d** Quantification of the mean normalized oIPSC amplitudes (time: *F*_(1.231,8.615)_ = 29.03 with Geisser–Greenhouse correction, *p* < 0.001, **p < 0.01, ***p < 0.001 by post hoc Dunnett compared to baseline recording condition). **e** Scheme of experimental approach to assess potential inhibitory synaptic connections between BNSTad and BNSTav neurons and their modulation by Y2R activation. BNSTad neurons were transduced using AAV-Syn-ChR2-eYFP and optogenetic experiments were performed 6 weeks later. **f** Localization of recorded neurons in BNSTav. **g** Time course of the mean normalized oIPSCs amplitudes at BNSTad to BNSTav synapses (*n* = 8/5). **h** Quantification of the mean normalized oIPSC amplitudes (time: *F*_(1.663,11.64)_ = 0.43 with Geisser–Greenhouse correction, *p* = 0.62). **i** Representative traces of sEPSC and sIPSC recorded subsequently from individual neurons in BNSTav and BNSTad in control and Cre-injected mice. **j** Quantification of excitation/inhibition ratios (calculated by sEPSC frequency/sIPSC frequency) in BNSTav and BNSTad in control and Cre-injected mice (BNSTav: control: *n* = 15/8, Cre: *n* = 14/3, BNSTad: control: *n* = 17/9, Cre: *n* = 15/7, *H*_(3)_ = 18.00, *p* < 0.001, *p < 0.05, **p < 0.01, ***p < 0.001 by post hoc Dunn. **k**, **l** Quantification of mean sEPSC amplitudes and frequencies. **m**, **n** Quantification of mean sIPSC amplitudes and frequencies.

Next, we tested the impact of Y2R modulation on interregional GABAergic connections between BNSTav and BNSTad using a corresponding experimental approach, but optogenetically activated a neuron in BNSTav and recorded oIPSCs in a BNSTad neuron or vice versa (Fig. 4a, b, e, f). While bath application of 200 nM PYY_3–36_ for 10 min led to a significant reduction in the mean oIPSC amplitudes of GABAergic inputs from BNSTav to BNSTad neurons (Fig. 4c, d), only a few GABAergic inputs from BNSTad to BNSTav neurons were Y2R-modulated (BNSTav to BNSTad: seven out of eight connected pairs, BNSTad to BNSTav: two out of eight connected pairs, *p* < 0.05, Fisher’s exact test), and the mean oIPSC amplitude of GABAergic inputs at BNSTad to BNSTav synapses did not change significantly upon PYY_3–36_ application (Fig. 4g, h). Results indicate that inter-regional inhibitory microcircuits are differentially regulated by local Y2R signaling, with stronger modulatory influence on BNSTav to BNSTad synapses compared to BNSTad to BNSTav synapses.

### Extrinsic GABAergic inputs to BNSTad are Y2R-modulated

Next, we tested whether extrinsic axonal inputs to the anterior BNST are regulated via Y2R. Postsynaptic inhibitory currents (eIPSCs) were evoked by a bipolar tungsten stimulation electrode placed dorsally to the recording electrode in the surrounding neuropil in slices from Cre-injected mice, assuming that local Y2R on BNST neurons are deleted (Supplementary Fig. 6A, B, E, F). Bath application of PYY_3–36_ had no effect on mean eIPSC amplitudes in BNSTav neurons (Supplementary Fig. 6C, D) while most extrinsic GABAergic inputs to BNSTad neurons were modulated by Y2R (BNSTad: 10 out of 13, BNSTav: 0 out of 6, *p* < 0.01, Fisher’s exact test) and mean eIPSC amplitudes were significantly reduced in BNSTad neurons (Supplementary Fig. 6G, H). These results corroborate specificity of our regional Y2R deletion approach and suggest a prevalent attenuating regulation of extrinsic GABAergic inputs to BNSTad via Y2R.

### Local Y2R control excitation/inhibition balance in BNSTav neurons

Considering the differential modulation of distinct intraregional, inter-regional and extraregional GABAergic circuits within the anterior BNST, we tested Y2R influences on network-driven excitation and inhibition balance in the anterior BNST. We recorded sEPSC and sIPSC in individual BNST neurons by voltage-clamping cells first close to the GABA (−65 mV), and then at the  glutamate (0 mV) reversal potential (Fig. 4i). To estimate the balance of excitation and inhibition within BNSTav and BNSTad, we calculated E/I ratios (sEPSC frequency/sIPSC frequency, Fig. 4j). Neither sEPSC amplitude and/or frequency nor sIPSC amplitude and/or frequency differed between experimental groups (Fig. 4k–n). However, only deletion of Y2R in BNSTav neurons significantly shifted E/I ratio in BNSTav towards greater inhibitory drive (Fig. 4i, j). These data suggest that endogenous NPY controls E/I balance in BNSTav neurons via local Y2R.

## Discussion

Fiber bundles of the stria terminalis and anterior commissure divide the BNST into anterior/posterior and dorsal/ventral sections [21]. The anterior BNST receives massive input from CeA and is well positioned to regulate stress responsiveness owing to its projections to paraventricular hypothalamic nucleus [22] and ventral tegmental area [13]. High levels of Y2R exist in CeA and various subregions of the anterior BNST [23], on both local circuit neurons and within the stria terminalis in association with GABAergic, NPY-negative projection neurons [9]. Here we show that targeting Cre to BNSTav for functional deletion of Y2R in Y2^lox/lox^ mice increases conditioned fear during recall and extinction learning and promotes remote recall of fear, whereas Y2R deletion in BNSTad or CeA has no such effect.

The lack of behavioral effect after Y2R deletion in CeA is in line with observations that BNSTav is sparsely innervated by Y2R-expressing neurons from centromedial amygdala, whereas dense inputs from centrolateral amygdala are devoid of Y2R [13, 24]. Previous findings on Y2R-mediated alteration of fear extinction in CeA [12] are difficult to compare because different training paradigms were used and conclusions relied on both functional deletion of Y2R and overexpression of NPY_3–36_ in CeA.

Here, deletion of Y2R in BNSTav led to higher freezing in extinction training and remote recall tests, even though no group differences existed in baseline freezing across all behavior, acquisition of fear, or by the end of extinction training. Pharmacological intervention largely mimics these behavioral effects in that a single application of Y2R antagonist in BNSTav increases recall of remote fear and delays extinction learning in both Y2^lox/lox^ (present study) and C57BL/6N mice [10]. Together, these data imply that mediating neurons and Y2R are localized within the BNSTav. Of note, targeting Cre to BNSTav of Y2^lox/lox^ had an additional effect on conditioned fear recall, implying an effect of chronic Y2R deletion on consolidation or retrieval of fear memory. Our conclusions are: (1) Y2R in BNSTav suppresses recall of conditioned and remote fear with a high degree of specificity that cannot merely be attributed to a state-like effect, and (2) local circuit neurons within BNSTav and synaptic targets in the local neuropil are the relevant mediators of these Y2R-mediated behavioral influences.

Locally restricted laser scanning photoactivation via ChR2 combined with targeted whole-cell patch-clamp recordings allowed us to reliably study intrinsic BNST microcircuits. Compared with conventional “bulk” field of view laser stimulation, this approach prevents direct activation of the recorded neuron. Postsynaptic responses occurred stimulus-locked with high fidelity and little onset latency jitter, and they were sensitive to GABA_A/B_ receptor antagonists, corroborating the monosynaptic GABAergic nature of these intrinsic BNST connections. GABAergic neurons account for the majority of the overall BNST population [25–27], and inhibitory connections act as a gating mechanism for BNST output and modulation of extrinsic inputs [28–34]. In keeping with this, functional deletion of Y2R in BNSTav resulted in a decrease in E/I ratio in BNSTav most likely by eliminating a tonically dampening NPY effect on inhibitory drive in intraregional BNSTav microcircuits. Y2R are expressed in local circuit neurons in BNST [9, 23], and activation of presynaptic Y2R reduces GABAergic signaling to BNSTav neurons [35–37], although their behavioral significance remained unknown. Our targeted approach revealed that local GABAergic neurons form monosynaptic connections in both BNSTav and BNSTad, and stimulation of presynaptic Y2R attenuates inhibitory signaling at these connections. All tested intraregional connections were Y2R-modulated, arguing against differential cell type specificity. GABAergic neurons in BNSTav and BNSTad are mutually interconnected [13, 32]. Contrary to the homogeneous Y2R modulation of intraregional GABAergic BNST microcircuits, our results indicate that inter-regional inhibitory microcircuits are differentially regulated by local Y2R signaling with stronger modulatory influence on BNSTav to BNSTad synapses compared to BNSTad to BNSTav synapses. Our conclusions are: (1) local GABAergic neurons form monosynaptic connections bearing presynaptic Y2R within and in-between BNSTav and BNSTad, and (2) Y2R stimulation at BNSTav connections is mandatory for Y2R-mediated facilitation of fear extinction and attenuation of remote fear return. Noteworthy BNSTav and BNSTad receive additional strong GABAergic innervation from extrinsic nuclei like CeA [13, 38, 39]. Our results suggest a prevalent attenuating regulation of extrinsic GABAergic inputs to BNSTad via Y2R, which however do not contribute to Y2R-mediated regulation of fear extinction and recovery.

Little experimental evidence exists with regards to the precise source of NPY acting on local BNST Y2Rs. In a series of electrophysiological in vitro studies, Walter and colleagues demonstrated properties of local NPY-positive neurons in the anterior BNST characterized by a persistently high state of excitability [19]. Provided this, it is plausible that these NPY-positive neurons remain readily responsive to behaviorally relevant afferent stimuli ultimately translating into neuronal plasticity during fear extinction and remote fear return. It should be noted too that the highest density of NPY inputs to the BNST originate in the AgRP neurons of the arcuate nucleus of the hypothalamus [40, 41] and any changes in the neuronal activity of these neurons lead to changes in NPY release as well.

**Fig. 5:**
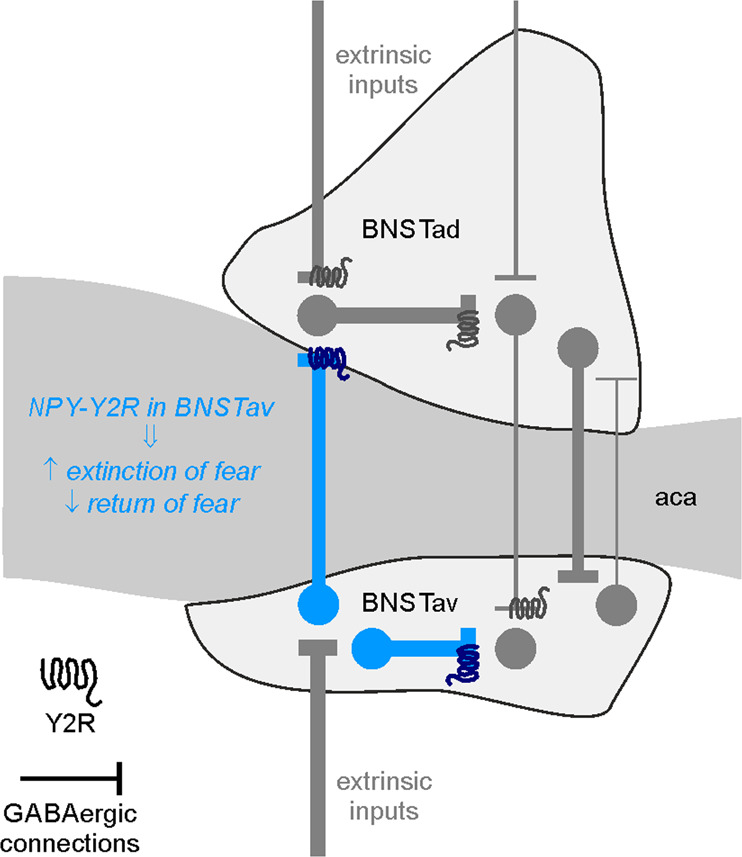
Schematic presentation of Y2R-modulated inhibitory BNST microcircuits facilitating extinction and reducing return of remote fear memories. In a simplified scenario, extinction training after threat exposure is a relevant stimulus for recruitment of the NPY-Y2R system in BNSTav (highlighted in blue), where stimulation of Y2R on GABAergic interneurons regulates the excitation/inhibition balance in local microcircuits promoting fear extinction and reducing return of fear. The thickness of arrows was adjusted to represent the relative incidence of respective connections (irrespective of Y2R-mediated modulation).

Correlation of synaptic network and behavioral function can be derived from activity patterns in optically identified types of neurons in BNSTav, where glutamatergic cells displayed enhanced activity during exposure to an aversive footshock or conditioned cue whereas GABAergic cells were inhibited [42], likely resulting in a marked increase in E/I ratio. Furthermore, chronic restraint stress increases NPY expression in BNST in DBA/2J mice [37, 43], and intranasal administration of NPY prior to or after stress exposure can attenuate or reverse PTSD-like behavior in rats, respectively [44, 45]. In human patients with PTSD, NPY plasma levels loosely parallel the disease course [9], implicating that NPY can act as a stress buffer in response to traumatic events in both rodents [46] and humans [47]. One study of a human single nucleotide polymorphism (SNP) which affects the expression of Y2Rs points to a correlation of increased Y2R expression to a decay in iconic memory processes [48]. Further studies are required however to draw a specific link between the Y2R gene polymorphisms in humans and their role in fear-related memory. Our study extends these conclusions by noting that extinction training after threat exposure is a relevant stimulus for recruitment of the NPY-Y2R system in BNSTav, where stimulation of Y2R on GABAergic interneurons regulates the E/I balance in local microcircuits (Fig. 5). The behavioral correlate is evident as facilitated extinction of a learned response to a threat and attenuation of its return at remote stages. Such a mechanism seems well suited to improve resilience against persistence of a threat response and symptomatologies associated with a dysregulated fear response system, such as PTSD.

## Supplementary information

Supplementary Information
