# Supplementary material for: Functional deletion of neuropeptide Y receptors type 2 in local synaptic networks of anteroventral BNST facilitates recall and increases return of fear
Source: Mol Psychiatry. 2020 Jul 24;26(7):2900–11. doi: 10.1038/s41380-020-0846-x (PMC8505243; doi:10.1038/s41380-020-0846-x)
Supplement: Supplementary file 1 — Supplementary Information [file 41380_2020_846_MOESM1_ESM.pdf]

Supplementary Information for

Functional deletion of neuropeptide Y receptors type 2 in local synaptic networks of anteroventral BNST facilitates recall and increases return of fear

Julia Constance Bartsch[1*] (Dr. med.), Sara Jamil[1*] (MSc), Jasmin Remmes[1] (Dr. rer. nat.), Dilip Verma[1§] (PhD), Hans-Christian Pape (Dr. rer. nat.) [1§†]

* J.C.B., S.J. shared first authorship in alphabetical order

§ D.V., H.C.P. shared senior authorship

Corresponding author: Hans-Christian Pape, Institute of Physiology I; Robert-Koch-Str. 27a, Westfälische Wilhelms-Universität Münster, D-48149 Münster; Germany

E-Mail: papechris@ukmuenster.de

Inventory of Supplementary Information

Supplementary Materials and Methods

- Behavioral Paradigm
- Viral infusion
- Verification of Cre-mediated recombination
- RNAscope- Fluorescence in situ hybridization of Y2R
- Electrophysiology

Supplementary Figures

- Supplementary Figure 1: Laser-assisted BNSTav and BNSTad dissection and schematic representation of Cre-recombination of Y2R.
- Supplementary Figure 2: Detection of Y2R mRNA in DAPI-positive nuclei of $Y2^{lox/lox}$ mice displays Cre-mediated deletion of Y2R within BNSTav.
- Supplementary Figure 3. Quantification of Y2R mRNA after Cre-mediated deletion of Y2R, in DAPI-positive nuclei within BNSTav of $Y2^{lox/lox}$ mice.
- Supplementary Figure 4: Pharmacological manipulation of Y2R within BNSTav and behavioral effects on acquisition, extinction and remote recall.
- Supplementary Figure 5: Cre-mediated deletion of Y2R within CeA and behavioral effects on acquisition, extinction and remote recall.
- Supplementary Figure 6: Extrinsic GABAergic inputs to BNSTad are Y2R-modulated.

Supplementary Materials and Methods

All behavioral experiments were performed in the light cycle.

Behavioral paradigm: On day 1, mice were presented with five CS+ (white noise, 30 sec, 80 dB), co-terminating with a footshock (US, 0.6 mA, 2 sec), and five CS- (2.5 kHz, 30 sec, 80 dB) with an inter-trial interval (ITI) varying 60-100 s in a TSE conditioning chamber. On day 2, mice were trained for extinction in a separate context (Macrolon cage Type III), through exposure to 5 CS- followed by 25 CS+ (duration 30 s, inter-stimulus interval 5 s). Fifteen days after conditioning, remote fear memory was assessed in the extinction context by presenting ten CS+ (30 s, inter-stimulus interval 5 s) [1]. Freezing (absence of movement except respiration > 1 s) was taken as behavioral expression of fear, recorded through two cameras controlled by MOVE software [2], scored online using a Key-logger and analyzed using a customized MATLAB script (MATLAB 12, The MathWorks, Natick, MA, USA) [3]. Freezing to the CS+ presentations was averaged across 3 consecutive CS presentations (3CS+ block).

Viral infusion: Viral transduction of neurons in control mice was performed using recombinant adeno-associated viruses of serotype 2 (rAAV2) carrying a fusion construct of the coding sequences of the light-activated Channelrhodopsin 2 and enhanced yellow fluorescent protein (eYFP) under the control of the human synapsin promoter (rAAV2-hSyn-hChR2-eYFP; University of North Carolina Vector Core, Chapel Hill, NC, USA). rAAV2s were produced using pAAV-hSyn-hChR2(H134R)-EYFP (gift from Karl Deisseroth, Addgene plasmid # 26973). For Cre-mediated deletion of Y2 receptors, adeno-associated viruses of hybrid-serotype 1/2 (AAV1/2) carrying Cre-hChR2(H134R)-eYFP under the control of the synapsin promoter were used (University Medical Center of the Johannes Gutenberg University Mainz,

Germany). In the latter construct, a 2A-mediated peptide cleavage linker was introduced between Cre and hChR2(H134R)-eYFP [4].

Verification of Cre-mediated recombination of Y2R by RT-PCR: Frozen brain sections of 16 µm thickness were cut on a cryostat at -20°C and placed on RNase-free PALM Membrane Slides (Carl Zeiss, Göttingen, Germany) coated with Poly-L-Lysine (Sigma-Aldrich, Taufkirchen, Germany). eYFP-labeled BNST (BNSTad and BNSTav) sections were dissected via laser microdissection (PALM MicroBeam system, Carl Zeiss, Bernried, Germany) (see Supplementary Figure 1). Isolated tissue probes were stored in lysis buffer (350 µl RLT buffer + 3.5 µl Mercapto + 5 µl carrier-RNA, Qiagen, Hilden, Germany) at -80 °C. RNA isolation was performed with DNase treatment using the RNeasyMicro-Kit (Qiagen). The RNA was eluted in 14 µl RNase free water and stored at -80 °C. cDNA synthesis was performed in a final volume of 11.5 µl by adding 5.5 µl of master mix [2.5 µl reverse transcriptase buffer, 1 µl DTT and 0.5 µl superscript reverse transcriptase III (Invitrogen, Karlsruhe, Germany), 0.5 µl dNTPs (Thermo-Fisher Scientific, Dreieich, Germany), 0.5 µl random Primers (Roche, Mannheim, Germany), 0.5 µl RNasin (Promega, Mannheim, Germany)], and 6 µl of RNA template. After incubation at 37°C for 1 h, cDNA samples were stored at −20°C until use. PCR amplification for Y2R recombination and GAPDH was performed with commercially available primers (mY2R recombination-forward 5′-AGCATCCAGAGAAGTGCAAC -3′, mY2R recombination-reverse 5′-TTAACATCAGCTGGCCTAGC-3′ [5]; GAPDH-forward 5′-TTGGCCGTATTGGGCGCCTGGTCA-3′, mGAPDH-reverse 5′-TGCCGTTGAATTTGCCGTGAGTGG-3′ (Eurofins Genomics, Ebersberg, Germany) using a standard PCR amplification protocol. In detail, to 3 µl of cDNA template, 1 µl of each respective reverse and forward primer (10 pmol/µl), 2 µl $MgCl_2$ (2.5 mM), 0.5

µl Taq polymerase (1.25 U, Promega, Mannheim, Germany) + dNTPs (Thermo-Fisher Scientific), 5 µl PCR buffer, and 7 µl distilled water were added. The amplification protocol consisted of an initial denaturation step of 3 min at 94°C, followed by 40 cycles for Y2R recombination (Denaturation: 94°C for 30 s, Annealing: 61°C for 60 s, Elongation: 72°C for 60 s) and 30 cycles for GAPDH (Denaturation: 94°C for 30 s, Annealing: 66°C for 60 s, Elongation: 72°C for 60 s), and finalized with a prolonged elongation step at 72°C for 7 min (Mastercycler gradient, Eppendorf, Hamburg, Germany). Amplified products were transferred to a 1.5% agarose gel and products separated by size (250 bp for Y2R recombination and 150 bp for GAPDH) in the agarose gel matrix along an electrical gradient for 35 min at 100 V in TAE-buffer (Merck). Fragments were visualized using Midori Green Advance (Biozym, Oldendorf, Germany). As a housekeeping gene, GAPDH served as a control for successful tissue collection and cDNA synthesis.

RNAscope- Fluorescence in situ hybridization of Y2R

In a separate set of $Y2^{lox/lox}$ mice, 3 animals per group were used to infuse control and Cre virus as mentioned in the Methods. After 3 weeks of post-surgical recovery, mice were anesthetized with isoflurane (5%) and rapidly decapitated. Brains were quickly removed, snap-frozen in isopentane and stored at -80°C. Detection of Y2R mRNA was performed in 16 µm thick slices using RNAScope Multiplex-Fluorescent assay kit with predesigned probes for mouse Y2R mRNA (RNAscope Probe- Mm-Npy2r; cat #315951-C3) following the manufacturer`s instructions (ACDbio, CA, USA). After final washes, the sections were incubated for 30 s with DAPI in order to stain the nuclei and mounted in Vectashield mounting medium (Vector Laboratories, Burlingame, CA, USA) and slides were stored at 4°C. Images were captured using a confocal microscope (Leica, Wetzlar, Germany) with predefined laser beam filter

settings for DAPI (cell nuclei) and Atto550 (Y2R). From BNSTav region, bilateral image stacks (1.5 µm steps) were acquired (2024x2024 resolution) in 2 coronal brain sections per animal using an HC PL APO CS2 20x/0.75 dry objective. Image stacks were merged into a 2D picture using maximum z-projection (mRNA expression) and used for further quantification with ImageJ 1.52p (Wayne Rasband, National Institute of Health, USA). After splitting the color channels, the DAPI signal was binarized, thresholded and dilated once (~40%), before applying the watershed function. DAPI-positive nuclei were detected using morphological segmentation plugin and automatically counted using the Analyze Particles function of ImageJ. Detected DAPI-positive nuclei were used as regions of interest (ROI) for the detection of Y2R mRNA signal ("dots"). Color channel "red" containing Y2R mRNA signal ("dots") was background corrected and converted into a binary signal. Y2R mRNA signal in overlaid ROIs was counted by using ImageJ cell counter plugin (at least 4-5 "dots" /DAPI-positive cell). Data is presented as percentage of DAPI-positive nuclei co-expressing Y2 mRNA in Supplementary Figure 3.

Electrophysiology: Mice were decapitated under deep isoflurane anesthesia (1-chloro-2,2,2-trifluoroethyl difluoromethyl ether; 2.5% in O_2; Abbot, Wiesbaden, Germany), and brains were quickly removed. Coronal slices (250 µm) containing the anterior BNST were obtained with a Leica VT1200S vibratome (Leica Microsystems CMS, Mannheim, Germany). The preparation was performed in ice-cold oxygenated (95% O_2, 5% CO_2), saccharose-based artificial cerebrospinal fluid (ACSF) containing (in mM): KCl 2.5, Na_2PO_4 1.25, PIPES 20, $CaCl_2$ 0.5, $MgSO_4$ 10, saccharose 200, glucose 10 at a pH of 7.35. After preparation, slices were kept under submerged conditions at 30°C for approximately 30 min and afterwards at room temperature in preincubation ACSF (in mM: NaCl 125, Na_2PO_4 1.25, $NaHCO_3$ 24, KCl 2.5, $CaCl_2$ 2, $MgSO_4$ 2, glucose 10 at a pH of 7.35).

Whole-cell patch-clamp recordings in voltage-clamp mode were obtained from BNST neurons under submerged conditions in physiological ACSF solution (in mM: NaCl 120, Na_2PO_4 1.25, $NaHCO_3$ 22, KCl 2.5, $CaCl_2$ 2, $MgSO_4$ 2, glucose 25 at a pH of 7.35) at room temperatures. In some experiments, postsynaptic inhibitory currents (eIPSCs) were evoked by a bipolar tungsten stimulation electrode placed dorsally to the recording electrode in the surrounding neuropil. 2-(3-Carboxypropyl)-3-amino-6-(4 methoxyphenyl) pyridazinium bromide (Gabazine, 10 µM), CGP55845 hydrochloride (2.5 µM) were added to the extracellular solution at the end of the experiment to verify the GABAergic nature of evoked postsynaptic currents. Patch-clamp electrodes (2-4 MΩ) were made of borosilicate glass (GC150T-10, Harvard Apparatus, Cambridge, UK) and filled with intracellular solution (in mM): Cs-methanesulfonate 135, CsCl 6, Mg-ATP 4, Na-GTP 0.4, Na_2-phosphocreatine 10, HEPES 10, EGTA 0.6 and had an osmolarity of 290–295 mOsm and pH of 7.2–7.3. Access resistance was monitored throughout the experiments and recordings with fluctuating access resistance were discarded. No series resistance compensation was used. Electrophysiological data were sampled at 10 kHz with an EPC10-double amplifier (HEKA, Lambrecht, Germany) and analyzed offline with Clampfit software (Molecular Devices Corporation, Sunnyvale, CA, USA). BNST neurons were clamped at a holding potential of 0 mV. After recording of stable baseline responses (stimulation frequency 0.05 Hz), PYY_{3-36} was bath-applied for 10 min at a flow rate of 3 ml/min and a concentration of 200 nM. For analysis, optogenetically or electrically evoked PSCs were normalized to baseline values. The mean of 9 (min 3 to 5) optogenetically activated or electrically evoked responses immediately before PYY_{3-36} application (baseline) was compared with 9 (min 13 to 15) evoked responses during PYY_{3-36} application (PYY) and 9 (min 23 to 25) responses after washout of PYY_{3-36} (wash). In figures, data points were binned from 3 consecutive responses when

illustrating the time course of IPSC amplitudes. Sample traces in Figure 3 are averages of 9 responses.

Detection and analysis of spontaneous excitatory and inhibitory postsynaptic currents (sEPSCs and sIPSCs) was done offline using Mini Analysis (Version 6.0.7, Synaptosoft, Fort Lee, NJ, USA). Detection threshold for amplitude of spontaneous events was set 3 times above root mean square (RMS)-baseline noise level. The baseline noise level was quantified from a recording episode without phasic synaptic currents using the RMS analysis routines of Mini Analysis. All events were visually controlled to exclude false-positive events. 3 min lasting recording episodes were analyzed.

Supplementary References

1 Verma D, Tasan R, Sperk G, Pape H-C. Neuropeptide Y2 receptors in anteroventral BNST control remote fear memory depending on extinction training. *Neurobiology of learning and memory* 2018; **149**: 144–153.

2 Meuth P, Gaburro S, Lesting J, Legler A, Herty M, Budde T *et al.* Standardizing the analysis of conditioned fear in rodents: a multidimensional software approach. *Genes, Brain and Behavior* 2013; **12**: 583–592.

3 Daldrup T, Remmes J, Lesting J, Gaburro S, Fendt M, Meuth P *et al.* Expression of freezing and fear-potentiated startle during sustained fear in mice. *Genes, Brain and Behavior* 2015; **14**: 281–291.

4 Donnelly ML, Luke G, Mehrotra A, Li X, Hughes LE, Gani D *et al.* Analysis of the aphthovirus 2A/2B polyprotein 'cleavage' mechanism indicates not a proteolytic reaction, but a novel translational effect: a putative ribosomal 'skip'. *J Gen Virol* 2001; **82**: 1013–1025.

5 Shi Y-C, Lin S, Wong IPL, Baldock PA, Aljanova A, Enriquez RF *et al.* NPY neuron-specific Y2 receptors regulate adipose tissue and trabecular bone but not cortical bone homeostasis in mice. *PLoS ONE* 2010; **5**: e11361.

Supplementary Figure 1: Laser-assisted BNSTav and BNSTad dissection and schematic representation of Cre-recombination of Y2R.

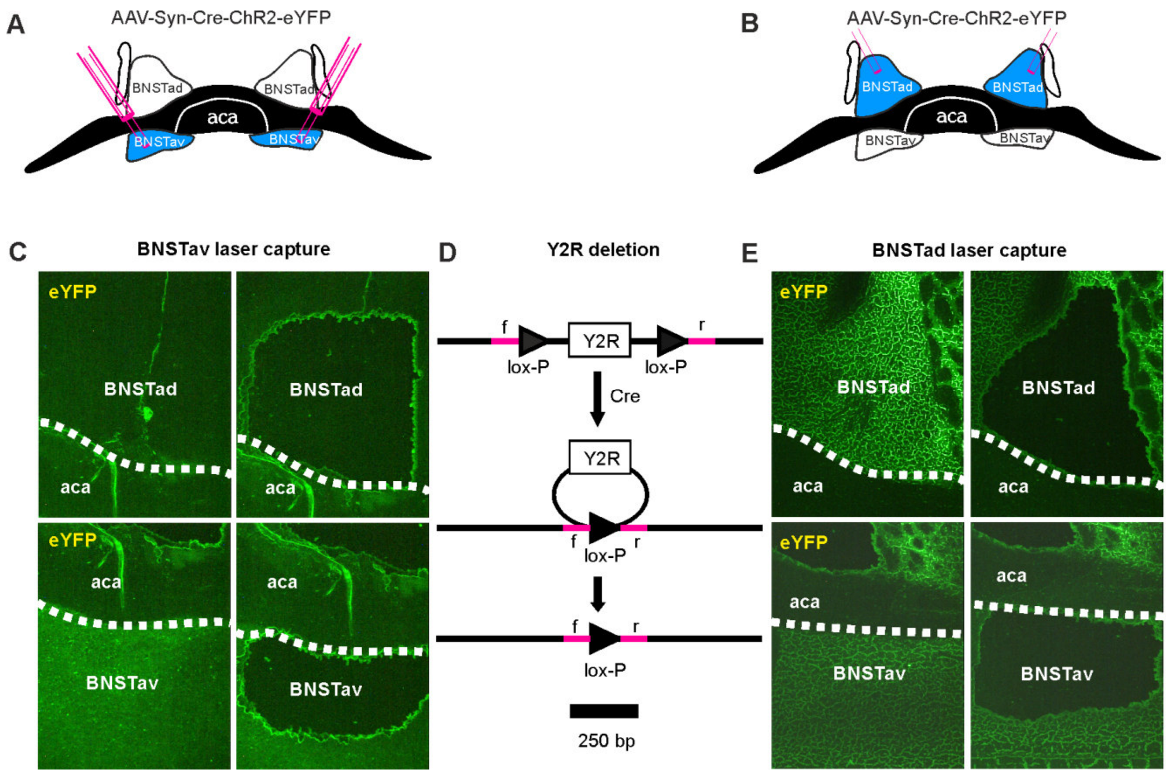

Schematic illustration of the local transfection site within BNSTav **(A)** and BNSTad **(B)**. Representative photomicrograph section from AAV-Syn-Cre-ChR2-eYFP-injected animals with local transfection in BNSTav **(C)** and BNSTad **(E)**. Laser-assisted excision of BNSTav and BNSTad subregions were performed in both cases and were used for RT-PCR. **(D)** Schematic diagram of Cre-mediated recombination of Y2R and the resulting 250 bp PCR product used to validate Cre-mediated deletion through standard PCR.

Supplementary Figure 2: Detection of Y2R mRNA in DAPI- positive nuclei of Y2$^{lox/lox}$ mice displays Cre-mediated deletion of Y2R within BNSTav.

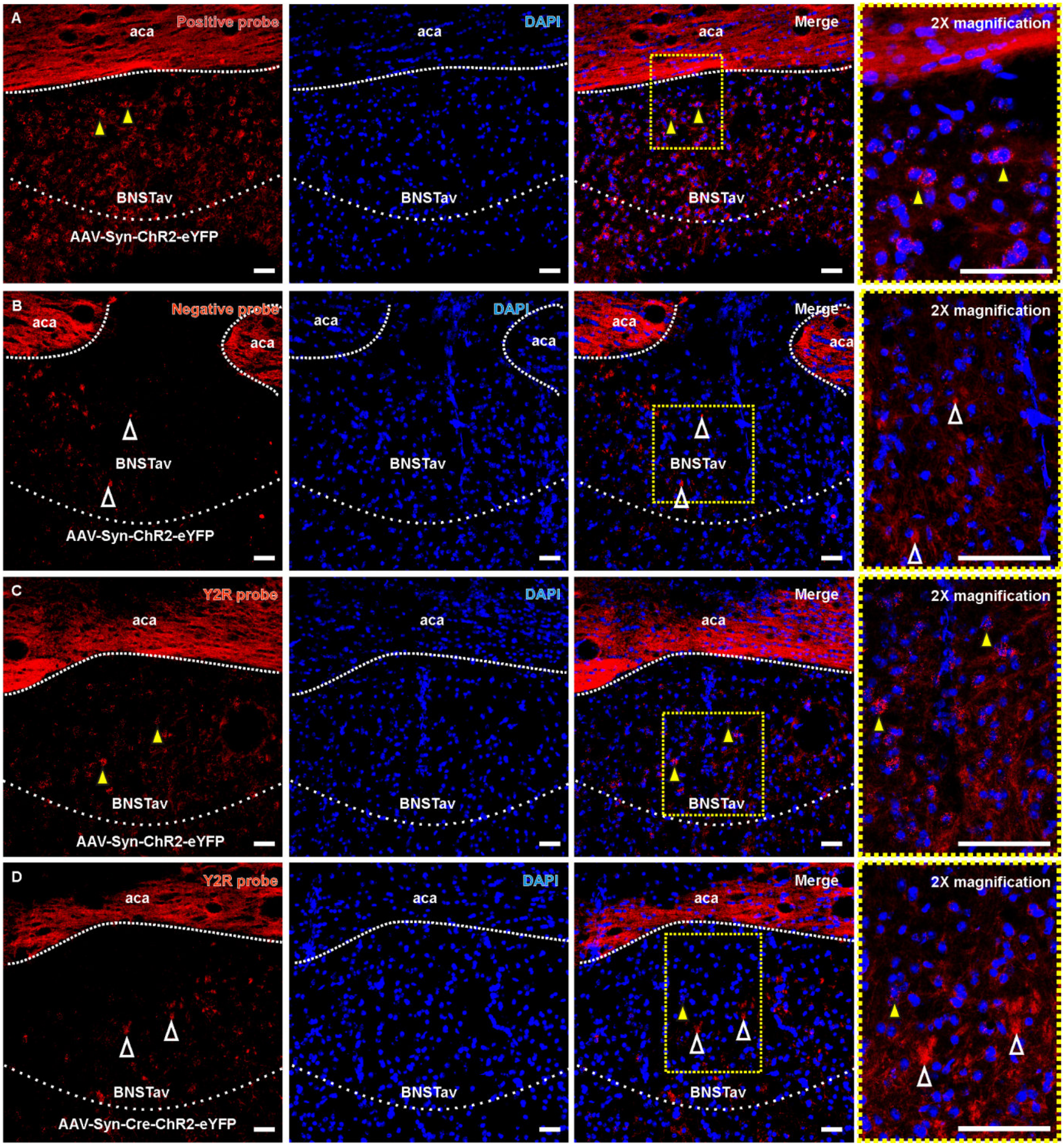

Row **(A)** and **(B)** shows positive and negative probe mRNA expression to validate the quality of fluorescence *in situ* hybridization (RNAscope) in BNSTav, and row **(C)** and **(D)** shows Y2R mRNA expression after control (AAV-Syn-ChR2-eYFP) and Cre (AAV-Syn-Cre-ChR2-eYFP) virus infusion, respectively. Following images in each of

the rows display the DAPI-positive nuclei within BNSTav, complete mRNA co-expression in DAPI-positive nuclei at 1x and 2 x magnification, respectively. Yellow arrows head highlight co-expression while empty white arrowheads depict background artifacts. DAPI (blue) demarcates cell nuclei. Scale bar A-D= 50 µm.

Supplementary Figure 3: Quantification of Y2R mRNA after Cre-mediated deletion of Y2R, in DAPI-positive nuclei within BNSTav of Y2$^{lox/lox}$ mice.

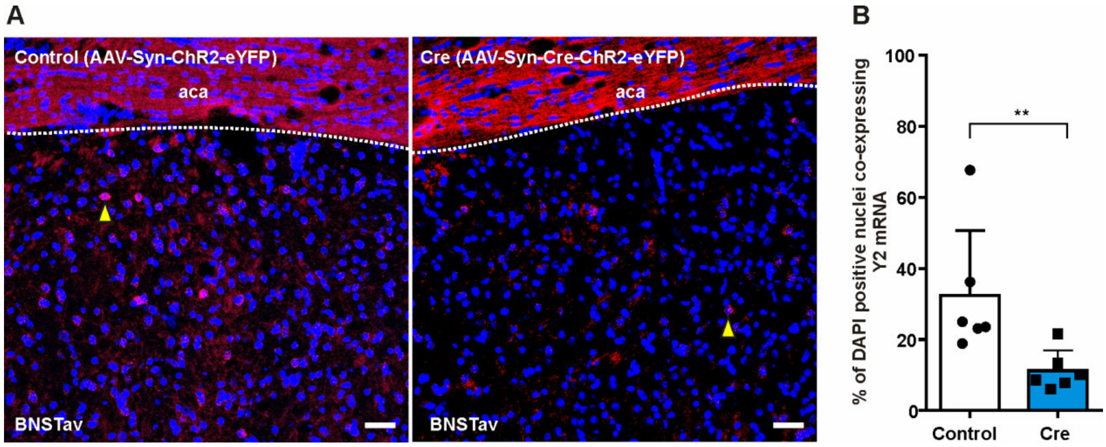

(A) Example of merged image demonstrates complete Y2R mRNA co-expression in DAPI-positive cells after control (AAV-Syn-ChR2-eYFP) and Cre (AAV-Syn-Cre-ChR2-eYFP) virus infusion. **(B)** Bar graph represents the percentage of DAPI-positive nuclei co-expressing Y2 mRNA. The percentage of DAPI-positive cells co-expressing Y2 mRNA was significantly reduced in Cre-injected mice (nonparametric Mann-Whitney test ** $p < 0.01$). DAPI (blue) demarcates cell nuclei. Scale bar = 50 µm.

Supplementary Figure 4: Pharmacological manipulation of Y2R within BNSTav and the behavioral effects on acquisition, extinction and remote recall.

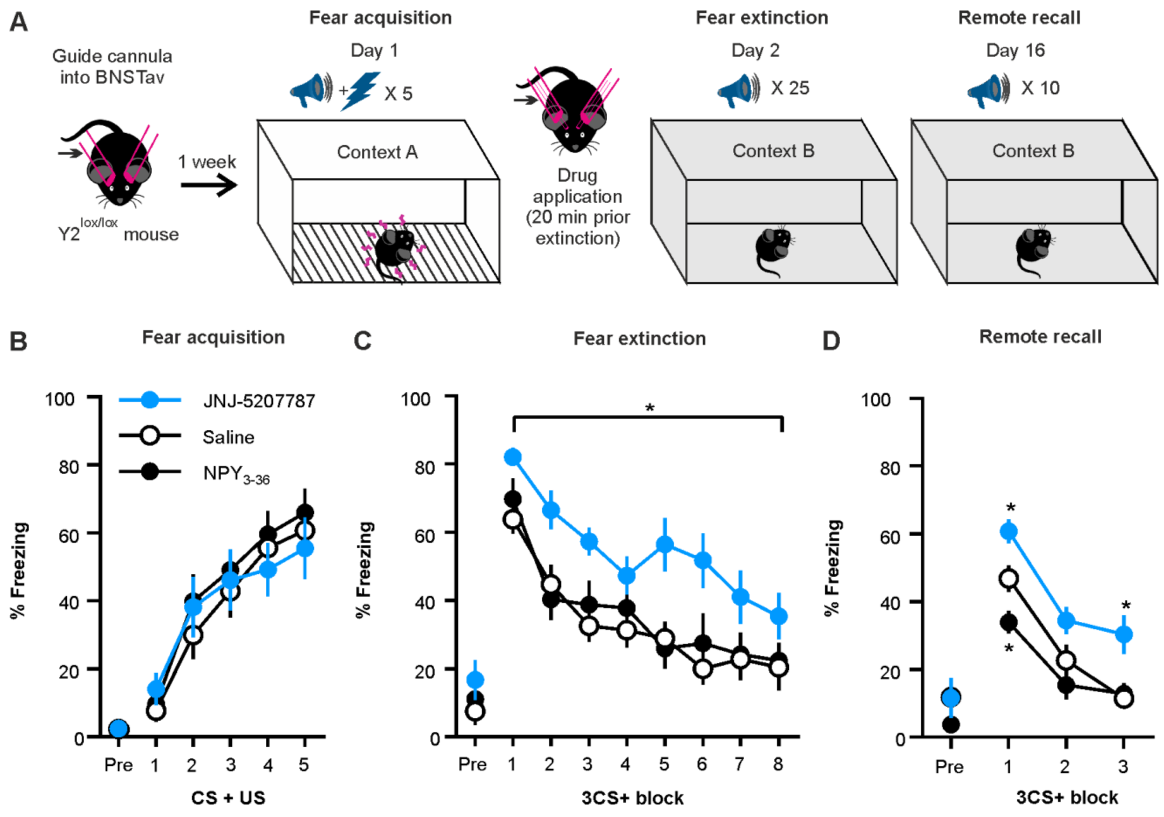

(A) Schematic representation of the experimental paradigm with timeline. **(B)** Statistical representation of fear acquisition to 5 CS + US presentations across time (group: $F_{(2, 27)}$ = 0.41, p = 0.662, partial η^2 = 0.030; time: $F_{(3.81,102.83)}$ = 84.58, p < 0.001, partial η^2 = 0.758, ε = 0.762; interaction: $F_{(7.61,102.83)}$ = 0.55, p = 0.806, partial η^2 = 0.039, ε = 0.762). **(C)** Statistical representation of fear extinction learning across time. Each data point represents freezing averaged across 3 consecutive CS+ blocks. Statistically significant group differences present with JNJ-injected mice showing a higher freezing level across time (8.58 ± 6.58%, p = 0.023, group: $F_{(2, 27)}$ = 4.58, p = 0.019, partial η^2 = 0.253; time: $F_{(8, 216)}$ = 47.40, p < 0.001, partial η^2 = 0.637; interaction: $F_{(16, 216)}$ = 1.29, p = 0.206, partial η^2 = 0.087). **(D)** Statistical representation of fear retrieval at remote time. Each data point represents freezing averaged across 3 consecutive CS+ blocks (group: $F_{(2, 27)}$ = 10.44, p < 0.001, partial

$\eta^2 = 0.436$; time: $F_{(3, 81)} = 68.81$, $p < 0.001$, partial $\eta^2 = 0.718$; interaction: $F_{(6, 81)} = 2.30$, $p = 0.042$, partial $\eta^2 = 0.146$). Significant differences in the freezing percentages between infusion groups during the first, second and the last 3CS+ block presentation ($F_{(2, 27)} = 13.92$, $p < 0.001$, partial $\eta^2 = 0.508$; $F_{(2, 27)} = 5.01$, $p = 0.014$, partial $\eta^2 = 0.271$; $F_{(2, 27)} = 7.57$, $p = 0.002$, partial $\eta^2 = 0.359$). During the 1st 3CS+ block, the NPY_{3-36}-infused $Y2^{lox/lox}$ mice have reduced level of freezing, while the JNJ-infused $Y2^{lox/lox}$ mice showed a higher freezing level as compared to control (NPY_{3-36}: -12.89 ± 4.94%, *$p = 0.015$; JNJ: 13.91 ± 5.16 %, *$p = 0.012$ by *post hoc* Bonferroni compared to control). At the 3rd 3CS+ block freezing levels were higher in JNJ-infused mice (JNJ: 18.90 ± 5.1 %, *$p < 0.005$ by *post hoc* Bonferroni compared to control).

Supplementary Figure 5: Cre-mediated deletion of Y2R within CeA and the behavioral effects on acquisition, extinction and remote recall.

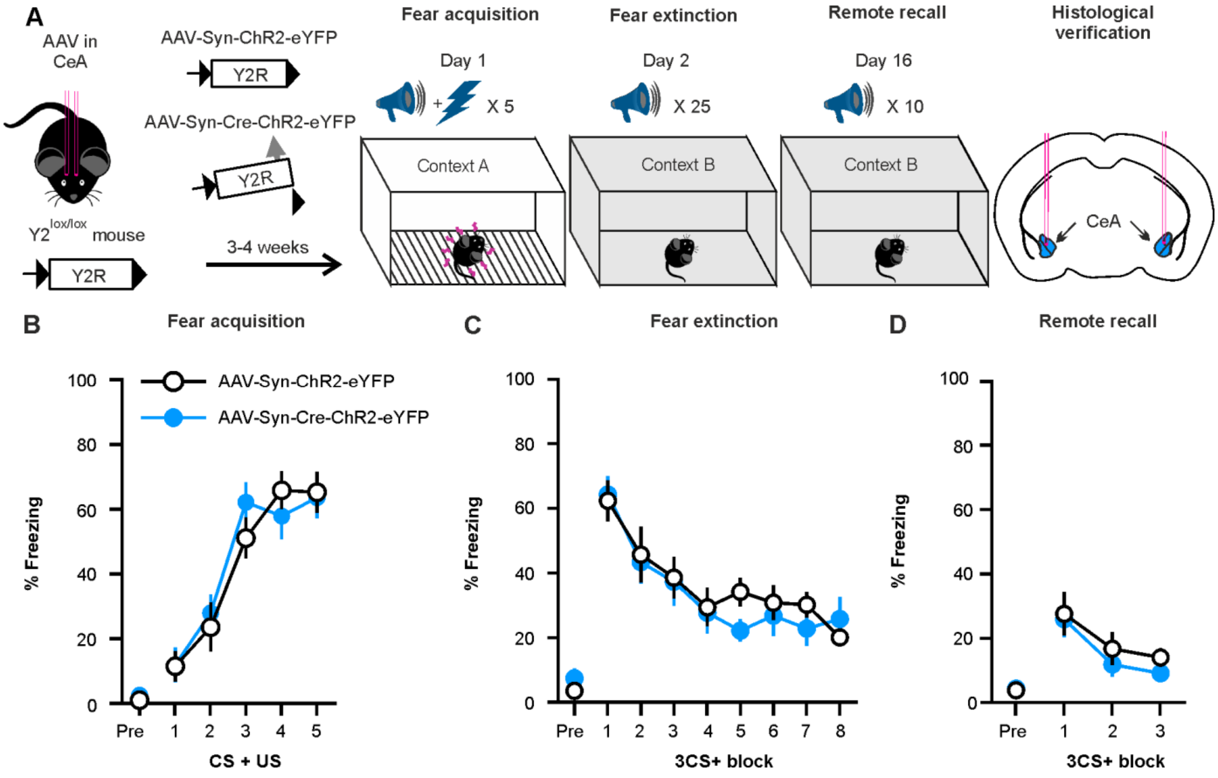

(A) Schematic representation of the experimental paradigm depicting the genetic model employed for Cre-mediated deletion of Y2R in CeA and the behavioral paradigm with timeline. **(B)** Statistical representation of fear acquisition across 5 CS + US presentations across time (group: $F_{(1, 12)}$ = 0.004, p = .953, partial η^2 < 0.001; time: $F_{(5, 60)}$ = 74.10, p = 0.001, partial η^2 = 00.861; interaction: $F_{(5, 60)}$ = 0.796, p = 0.557, partial η^2 = 0.062). **(C)** Statistical representation of fear extinction learning across time. Each data point represents freezing averaged across 3 consecutive CS+ blocks (group $F_{(1,12)}$ = 93.59, p = 0.880, partial η^2 = 0.002; time: $F_{(8, 96)}$ = 26.18, p < 0.001, partial η^2 = 0.686; interaction: $F_{(8, 96)}$ = 0.87, p = 0.55, partial η^2 = 0.067). **(D)** Statistical representation of fear retrieval at remote time. Each data point represents freezing averaged across 3 consecutive CS+ blocks (group: $F_{(1, 27)}$ = 0.27, p = 0.605,

partial $\eta^2 = 0.010$; time: $F_{(3, 81)} = 38.38$, $p < 0.001$, partial $\eta^2 = 0.587$; interaction: $F_{(3, 81)} = 1.19$, $p = 0.570$, partial $\eta^2 = 0.024$).

Supplementary Figure 6: Extrinsic GABAergic inputs to BNSTad are Y2R-modulated.

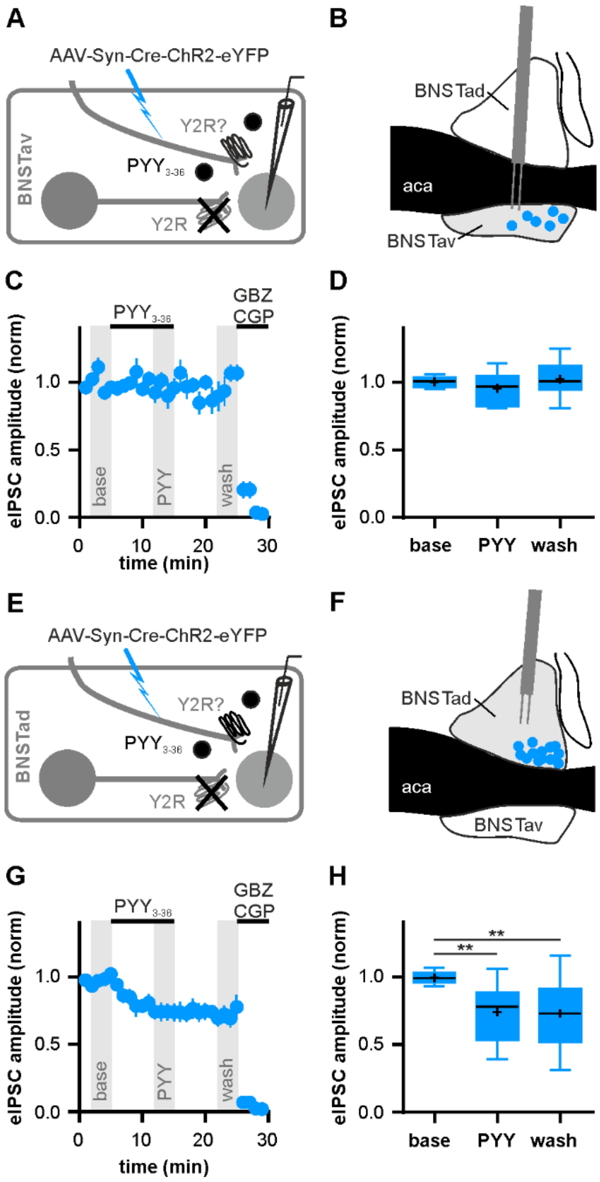

(A) Scheme of experimental approach to assess potential extrinsic inhibitory synaptic connections to BNSTav neurons and their modulation by Y2R activation. BNSTav neurons were transduced using AAV-Syn-Cre-ChR2-eYFP and optogenetic experiments were performed six weeks later. **(B)** Localization of recorded neurons in BNSTav. **(C)** Time course of the mean normalized eIPSCs amplitudes at extrinsic inputs to BNSTav neurons (n = 6/3). **(D)** Quantification of the mean normalized eIPSC amplitudes (one-way RM ANOVA with Greenhouse-Geisser correction, factor

time: $F_{(1.337, 6.683)}$ = 0.84, p = 0.43). **(E)** Scheme of experimental approach to assess potential extrinsic inhibitory synaptic connections to BNSTad neurons and their modulation by Y2R activation. BNSTad neurons were transduced using AAV-Syn-Cre-ChR2-eYFP and optogenetic experiments were performed six weeks later. **(F)** Localization of recorded neurons in BNSTad. **(G)** Time course of the mean normalized eIPSCs amplitudes at extrinsic inputs to BNSTad neurons (n = 13/8). Y2R activation reduced eIPSC amplitudes at extrinsic inputs to BNSTad neurons in Cre-injected mice. **(H)** Quantification of the mean normalized eIPSC amplitudes (one-way RM ANOVA with Greenhouse-Geisser correction, factor time: $F_{(1.912, 22.94)}$ = 10.35, p = 0.0007, ** $p < 0.01$ by *post hoc* Dunnett compared to baseline recording condition).
